# The association of serum Klotho concentrations with hyperlipidemia prevalence and lipid levels among US adults: a cross-sectional study

**DOI:** 10.1186/s12889-023-16566-y

**Published:** 2023-08-28

**Authors:** Shunli Jiang, Yongxin Wang, Zengliang Wang, Lu Zhang, Feng Jin, Bo Li

**Affiliations:** 1 Department of Public Health, Jining Medical University, Rencheng District, #33 Jianshe RoadShan Dong, Jining, 272000 China; 2 Department of Neurosurgery Center, The First Affiliated Hospital of Xinjiang Medical University, Urumqi, Xinjiang China; 3 Department of Neurosurgery, Affiliated Hospital of Jining Medical University, Jining, China

**Keywords:** Klotho, Hyperlipidemia, Triglycerides, Lipid, Cross-sectional study

## Abstract

**Background:**

Klotho has anti-oxidative and anti-inflammatory properties. However, little is known about whether high Klotho concentrations were associated with reduced hyperlipidemia risk and improved plasma lipid levels.

**Methods:**

Participants with complete data on serum Klotho and plasma lipid concentrations from the 2007–2016 National Health and Nutrition Examination Survey were included. Weighted regression models were fitted to explore the association of Klotho concentrations with hyperlipidemia risk and plasma lipid levels while restricted cubic spline models were applied to explore the dose–response relationship. Additionally, we assessed the mediating effects of C-reaction protein (CRP) on the foregoing association.

**Results:**

Individuals in the fourth and fifth quintile of serum Klotho had an adjusted odds ratio (OR) of 0.77 (95%CI: 0.65, 0.93) and 0.67 (95%CI: 0.65, 0.93) for hyperlipidemia. Doubling of serum Klotho concentrations was associated with decreased hyperlipidemia risk (OR = 0.81; 95%CI: 0.68, 0.95) and triglyceride levels (13.25 mg/dL; 95%CI: 4.02, 22.47), with a monotonic dose–response relationship. Individuals in the fourth and fifth quintile of serum Klotho had a 0.07 (95%CI: 0.002, 0.13), 0.08 (95%CI: 0.02, 0.15) and 0.05 (95%CI: –0.03, 0.12) mg/dL decreased CRP levels, with a marginally significant trend (*P*_trend_ = 0.05).

**Conclusions:**

Higher Klotho concentrations were associated with reduced hyperlipidemia risk and triglyceride levels. Klotho supplementation maybe a promising method to intervene and prevent hyperlipidemia, but the underlying mechanism should be further explored.

**Supplementary Information:**

The online version contains supplementary material available at 10.1186/s12889-023-16566-y.

## Introduction

Hyperlipidemia, particularly elevated plasma triglyceride (TG) and low-density lipoprotein cholesterol (LDL-C), is the most encountered lipid abnormalities. Hyperlipidemia is pivotal to cardiovascular disease, stroke, non-alcoholic fatty liver disease (NAFLD), and acute pancreatitis [1, 2]. Hyperlipidemia have been identified as a severe challenge in most high-income countries, its prevalence now is rapidly increasing even in low-incoming settings. Globally, high LDL-C caused 3.00 million deaths in 1990 and 4.40 million deaths in 2019 [3]. From 1990 to 2019, the disability-adjusted life of years caused by high LDL-C increased by 41% [3]. Besides, nearly half of deaths caused by IHD (8.54 million) in 2019 were due to high LDL-C levels (3.78 million) [4]. Hyperlipidemia is caused by a mixture of multiple genetic variations and environmental factors including obesity, diabetes, and unhealthy lifestyles [4]. The two mainstays for management of hyperlipidemia are lifestyle changes (dietary modification, exercise, weight loss, reduction of alcohol intake) and drug treatment (statins, fibrates, niacin) [5]. To alleviate the disease burden of hyperlipidemia, there exists an unfulfilled need for more efficacious interventions to reduce TG and LDL-C levels within the population.

The *Klotho* gene, renowned as an aging suppressor gene, is discovered by Makoto Kuro-o in 1997 [6]. It encodes a transmembrane protein that is mostly expressed in the kidneys. Mice lacking the gene presented premature aging and shortened lifespan, whereas its overexpression largely extended the lifespan [7]. There are two forms of Klotho: membrane-bound Klotho (mKlotho) and soluble Klotho (sKlotho). mKlotho has two forms: αKlotho and βKlotho. αKlotho acts as a co-receptor for FGF23 to handle phosphate and mineral homeostasis, whereas βKlotho functions as the primary receptors for FGF21 and FGF19, which respectively regulate metabolism during fasting and feeding [7–9]. The Klotho-FGF endocrine system holds significant importance in the development of age-related illnesses such as hypertension, diabetes, chronic kidney disease, arteriosclerosis, and cancer [10, 11]. sKlotho is present in the blood, cerebrospinal fluid, and urine where it can function as a circulating hormone. sKlotho exerts a multitude of beneficial effects including anti-inflammatory and anti-oxidant properties, regulation of ion channels, improving glucose metabolism, suggesting potential therapeutic applications in treating various diseases [12, 13].

Studies concerning the association of Klotho with hyperlipidemia prevalence and lipid concentrations are scarce and inconsistent. Kobayashi et al. indicated that hepatocyte βKlotho could regulate lipid homeostasis via suppressing the synthesis of bile acid [14]. Rao et al. observed reduced lipid storage in both the liver and adipose tissue after administration of αKlotho in obese mice [15]. Dongiovanni et al. noticed that βKlotho had a defensive function in safeguarding hepatocytes against lipotoxicity and inflammation in hepatocytes [16]. However, Kamari et al. discovered that there was a positive correlation between Klotho concentrations and plasma cholesterol levels while its active domain may have a favorable effect on plasma TG levels [17]. The benefits effects of sKlotho on blood pressure, glucose regulation, and lipid profile were suggested in some epidemiological studies [18, 19]. In addition, the association between FGF21 and lipid homeostasis is still far from consistent [20–22]. Herein, we conducted a cross-sectional study to examine serum Klotho levels in relation to the prevalence of hyperlipidemia, as well as lipid concentrations, in U.S. adults.

## Methods

### Study population

The National Health and Nutrition Examination Survey (NHANES) is conducted by the Centers for Disease Control and Prevention (CDC) in the United States. The health and nutritional status of the non-institutionalized population was assessed by interviews, physical examinations, and laboratory tests via a complex, multistage, probability sampling design [23]. The study design, protocol, data collection and analysis procedures have been reported in detail [24]. The study protocol was approved by the research ethics review board of the National Center for Health Statistics (NCHS) of the CDC, and all participants provided written informed consent. Our study conformed to the guideline for strengthening the reporting of observational studies in epidemiology statement [25].

Participants between the ages of 40 and 79 from NHANES 2007–2008 to 2015–2016 cycles were included in the study (*n* = 17,389). Serum Klotho concentrations were only measured in this specific age group and cycles. Participants with no missing data on serum Klotho and plasma lipid concentrations were included in the analysis (*n* = 13,764). Data on demographics, physical examinations, and laboratory tests were gathered during in-home interviews and study visits conducted at a mobile examination center (MEC) [26]. Medication use was obtained through the interviewer’s observation of individual’s prescription medications.

### Serum Klotho levels

Serum sKlotho concentrations were measured by a commercial ELISA kit (IBL International, Japan). As per the manufacturer’s instructions, the analysis of each sample was carried out twice, and the average of the two results was used as the final value. Each plate was analyzed with two quality control samples containing low and high concentrations of Klotho to ensure accuracy. Sample analyses were repeated when the assigned value of a quality control sample exceeded two standard deviations. The sensitivity of the assay was 4.33 pg/mL, with both the intra-assay and inter-assay coefficient of variation was under 5%. The detail description of laboratory methodology is available on the website at https://wwwn.cdc.gov/Nchs/Nhanes/2007-2008/SSKL_E.htm.

### Lipid concentrations and hyperlipidemia diagnosis

Lipid concentrations including fasting TG, Total cholesterol (TC), high-density lipoprotein cholesterol (HDL-C), and LDL-C were determined through laboratory measurement. LDL-C was calculated according to the Friedewald equation (LDL-C = TC – HDL-C – TG/5). Hyperlipidemia was defined as TG ≥ 150 mg/dL, TC ≥ 200 mg/dL, HDL-C < 40 mg/dL in males (50 mg/dL in females), LDL-C ≥ 130 mg/Dl, or current use of anti-hyperlipidemic medications [27, 28].

### Covariates

During the household interview, the computer-assisted personal interviewing system was used to collect sociodemographic data including age, sex, race/ethnicity, education, income, smoking status, and medical conditions. Self-reported race/ethnicity was classified into non-Hispanic black, non-Hispanic white, Mexican American, and other race (including multi-racial and other Hispanic group). Educational achievement was divided into three categories: did not complete high school, graduated high school or obtained a General Educational Development (GED) certificate, and attended college or beyond. Income levels were categorized into three tiers based on the family’s income-to-poverty ratio (IPR): less than 1.30, 1.30 to 3.49, or 3.50 or greater. The smoking status was divided into three categories: never smokers, former smokers, and current smokers (those who have smoked at least 100 cigarettes in their lifetime and continue to smoke). The alcohol questionnaire is administered during the physical exam at the MEC. The drinking status was classified into three categories: never drinkers, former drinkers, and current drinkers (who have consumed at least 12 alcoholic beverages in their lifetime and still continue to consume alcohol).

Hypertension was defined as SBP ≥ 140 mm Hg, DBP ≥ 90 mm Hg, a self-reported physician diagnosis, or currently taking antihypertensive medications [29]. Diabetes mellitus was defined as hemoglobin A1c ≥ 6.5%, fasting plasma glucose ≥ 7.0 mmmol/L, random plasma glucose or 2 h plasma glucose (oral glucose tolerance test) ≥ 11.1 mmmol/L, a self-reported physician diagnosis, or current use of antidiabetic medications or insulin [30]. The Charlson Comorbidity Index (CCI), which considered the number and seriousness of pre-defined comorbid conditions, was a reliable and valid tool that could accurately evaluate clinical situations and predict long-term mortality [31]. Thus, the CCI was used to access physical condition and avoid the multicollinearity among comorbidities. Estimated glomerular filtration rate (eGFR) was determined using the CKD-EPI creatinine equation [32].

### Statistical analysis

As recommended by the NCHS, the MEC exam weight, stratum, and PSU variables were incorporated into our analyses to account for the complex study design. Comparisons of participants’ characteristics, serum Klotho and plasma lipid concentrations between the hyperlipidemia and non-hyperlipidemia group were performed using the Student’s *t*-test, Mann–Whitney *U* test, and chi-square test for different types of variables.

Logistic regression models were employed to evaluate the odds ratio (OR) and 95% confidence interval (CI) of serum Klotho concentrations for the association with hyperlipidemia risk in the “survey” R package. Multivariable linear regression models were utilized to evaluate the coefficients and 95% CI linking serum Klotho concentrations to TG, TC, HDL-C, and LDL-C levels. The regression model incorporated both categorical and continuous models. The categorical model categorized serum Klotho concentrations into quintiles and analyzed the linear trend using the median value of each quintile as a continuous variable. Meanwhile, the continuous model logarithmically transformed serum Klotho concentrations to mitigate the impact of extreme values. Covariates were a priori selected based on biological plausibility and prior empirical evidence. They have the potential to confound the relationship between Klotho and related health conditions. Model 1 was the crude model. Model 2 accounted for inherent factors such as age, sex, and race/ethnicity. Model 3 further adjusted for confounding variables including educational attainment, IPR, BMI, smoking and drinking status, hypertension, diabetes mellitus, CCI, eGFR, and energy intake. In the “rms” R package, a weighted restricted cubic spline (RCS) model was constructed to examine the dose–response relationship between serum Klotho concentrations and prevalence of hyperlipidemia, along with plasma lipid levels.

Subgroup analyses were performed to examine whether potential confounding variables such as age, sex, race/ethnicity modify the effect of serum Klotho concentrations on hyperlipidemia prevalence. Interaction effects were evaluated by incorporating a multiplicative interaction term between serum Klotho concentrations and the stratification variable. Inflammation is one of the pathological mechanisms of hyperlipidemia, and Klotho may alleviate hyperlipidemia by exerting its anti-inflammatory effects. Hence, we assessed the mediating role of C-reaction protein (CRP) on the relationship of serum Klotho concentrations with hyperlipidemia prevalence and plasma lipid levels. Sensitivity analyses were conducted to verify the robustness of our findings. Firstly, to minimize the potential impact of extreme values, we separately repeated analyses after: a) excluding participants with serum Klotho concentrations that were higher or lower than the mean ± 3 × standard deviation; b) excluding participants with extremely high or low BMI levels (< 15 or ≥ 40 kg/m2) or a CCI score of 5 or higher. Secondly, taking anti-hyperlipidemic drugs might cover the potential beneficial effect of Klotho on hyperlipidemia, thus we repeated the regression models after excluding individuals under anti-hyperlipidemic treatment. All these data analyses were performed using the R software (version 4.2.1).

## Results

### Basic characteristics

Among 13,764 adults aged 40–79 years (mean 56), 80.0% were diagnosed with hyperlipidemia (Table 1). There was a significant difference in the mean age between the hyperlipidemia group (mean 57) and the non-hyperlipidemia group (mean 53) (*P* < 0.001). Participants with hyperlipidemia had higher BMI and CCI compared to other participants. Additionally, participants with hyperlipidemia had lower educational attainment, eGFR, and energy intake. Moreover, this group was found to have a higher likelihood of being Non-Hispanic white, smokers, and former drinkers, as well as being predisposed to hypertension and diabetes mellitus.

**Table 1:** Basic characteristics of the study participants, NHANES 2007–2015

Characteristics	Total population (*n* = 13,764)	No-Hyperlipidemia (*n* = 2755)	Hyperlipidemia (*n* = 11,009)	*P* value
Age, mean (SE), year	56.2 ± 0.2	53.3 ± 0.3	56.9 ± 0.2	** < 0.001**
Sex				0.079
Female	7097 (51.6)	1307 (50.5)	5790 (52.7)	
Male	6667 (48.4)	1448 (49.5)	5219 (47.3)	
Race/ethnicity				** < 0.001**
Non-Hispanic black	2727 (19.8)	741 (12.9)	1986 (8.3)	
Non-Hispanic white	5920 (43.0)	1059 (68.9)	4861 (73.9)	
Mexican American	2188 (15.9)	423 (7.2)	1765 (6.6)	
Other	2929 (21.3)	532 (11.1)	2397 (11.3)	
BMI, kg/m^2^				** < 0.001**
< 25	3607 (27.1)	1055 (40.9)	2552 (25.1)	
25–30	4852 (36.4)	910 (34.6)	3942 (37.4)	
≥ 30	4869 (36.5)	720 (24.6)	4149 (37.5)	
IPR				0.500
< 1.30	3886 (30.8)	759 (18.3)	3127 (17.7)	
1.30–3.49	4529 (35.9)	887 (31.4)	3642 (33.1)	
≥ 3.50	4213 (33.4)	883 (50.3)	3330 (49.3)	
Educational level				**0.008**
Less than high school	1876 (13.6)	321 (5.8)	1555 (6.4)	
High school or GED	5062 (36.8)	981 (29.9)	4081 (33.2)	
College or above	6818 (49.6)	1452 (64.3)	5366 (60.4)	
Smoking status				** < 0.001**
Never smokers	7074 (51.4)	1499 (56.5)	5575 (50.5)	
Former smokers	3986 (29.0)	701 (26.2)	3285 (30.7)	
Current smokers	2697 (19.6)	554 (17.3)	2143 (18.8)	
Drinking status				** < 0.001**
Never drinkers	1870 (14.7)	344 (10.0)	1526 (10.8)	
Former drinkers	2795 (21.9)	475 (14.6)	2320 (19.1)	
Current drinkers	8088 (63.4)	1713 (75.5)	6375 (70.1)	
Hypertension				** < 0.001**
No	6326 (46.0)	1590 (64.7)	4736 (48.0)	
Yes	7438 (54.0)	1165 (35.3)	6273 (52.0)	
Diabetes				** < 0.001**
No	10,195 (74.1)	2341 (90.1)	7854 (78.0)	
Yes	3560 (25.9)	410 (9.9)	3150 (22.0)	
CCI				** < 0.001**
< 1	5543 (40.3)	1402 (53.9)	4141 (40.3)	
≥ 1	8221 (59.7)	1353 (46.1)	6868 (59.7)	
eGFR, mL/min/1.73 m^2^				** < 0.001**
< 60	1345 (9.8)	175 (4.7)	1170 (8.9)	
60–90	5777 (42.0)	1051 (40.2)	4726 (45.9)	
≥ 90	6637 (48.2)	1527 (55.1)	5110 (45.2)	
Klotho, pg/mL	797.6 (656.5, 979.5)	835.4 (676.2, 1025.4)	789.6 (651.0, 971.2)	** < 0.001**
TG, mg/dL	109.0 (76.0, 162.0)	73.0 (57.0, 97.0)	123.0 (87.0, 176.0)	** < 0.001**
TC, mg/dL	198.0 (172.0, 226.0)	178.0 (164.0, 189.0)	208.0 (177.0, 234.0)	** < 0.001**
HDL-C, mg/dL	51.0 (42.0, 63.0)	58.0 (50.0, 67.0)	49.0 (40.0, 62.0)	** < 0.001**
LDL-C, mg/dL	116.0 (94.0, 141.0)	100.0 (87.0, 112.0)	124.0 (98.0, 147.0)	** < 0.001**
Energy intake, kcal	1937.5 (1511.5, 2445.5)	2014.5 (1595.5, 2529.5)	1920.0 (1489.0, 2424.5)	** < 0.001**

*Abbreviations*: *SE* Standard error, *BMI* Body mass index, *IPR* Family income to poverty ratio, *GED* General Educational Development, *CCI* Charlson Comorbidity Index, *eGFR* Estimated glomerular filtration rate, *TG* Triglycerides, *TC* Total cholesterol, *HDL-C* High-density lipoprotein cholesterol, *LDL-C* Low-density lipoprotein cholesterol

### Serum Klotho distribution

As shown in Table 1, the median value of serum Klotho was 797.6 pg/mL in the total population. The median serum Klotho value in the hyperlipidemia group (789.6 pg/mL) was significantly lower compared to the non-hyperlipidemia group (835.4 pg/mL) (*P* < 0.001). As indicated in Table 2, serum Klotho concentrations demonstrated significant elevation in youngers, females, Non-Hispanic black, non-smokers, non-drinkers, and individuals with low BMI, low CCI, or high eGFR than in their corresponding reference groups (all *P* < 0.05).

**Table 2:** Distributions of serum Klotho distribution and odds ratio (95% confidence interval) for hyperlipidemia in different subgroups

**Variables**	**N (%)**	**Concentrations**		hyperlipidemia	
		**Median (25th-75th)**	***P* value**	**OR (95%CI)**	***P***_**interaction**_
Age (year)					
< 58	6844 (49.7)	813.5 (669.6, 1001.9)	** < 0.001**	0.84 (0.68, 1.04)	0.687
≥ 58	6920 (50.3)	777.7 (636.1, 955.2)		**0.74 (0.61, 0.90)**	
Sex					
Female	7097 (51.6)	811.6 (660.2, 1005.9)	** < 0.001**	**0.79 (0.63, 0.99)**	0.546
Male	6667 (48.4)	783.6 (651.6, 957.1)		0.84 (0.66, 1.05)	
Race/ethnicity					
Non-Hispanic black	2727 (19.8)	832.0 (654.5, 1087.8)	** < 0.001**	**0.70 (0.57, 0.87)**	0.877
Non-Hispanic white	5920 (43.0)	790.6 (654.6, 967.8)		0.82 (0.65, 1.04)	
Mexican American	2188 (15.9)	810.4 (658.3, 988.3)		0.78 (0.58, 1.04)	
Other	2929 (21.3)	818.9 (667.8, 999.7)		0.85 (0.63, 1.16)	
BMI, kg/m^2^					
< 25	3607 (27.1)	810.4 (671.5, 1000.3)	** < 0.001**	0.85 (0.66, 1.09)	0.403
25–30	4852 (36.4)	787.5 (645.0, 965.1)		0.83 (0.62, 1.11)	
≥ 30	4869 (36.5)	796.9 (654.5, 980.5)		**0.73 (0.57, 0.94)**	
IPR					
< 1.30	3886 (30.8)	799.3 (651.1, 988.3)	0.726	**0.71 (0.55, 0.94)**	0.874
1.30–3.49	4529 (35.9)	794.4 (652.4, 977.7)		0.87 (0.65, 1.15)	
≥ 3.50	4213 (33.4)	800.7 (661.3, 977.1)		0.80 (0.63, 1.02)	
Educational level					
Less than high school	1876 (13.6)	800.4 (662.0, 981.1)	**0.030**	1.01 (0.69, 1.49)	0.592
High school or GED	5062 (36.8)	790.3 (642.9, 965.1)		0.84 (0.64, 1.10)	
College or above	6818 (49.6)	801.1 (662.3, 985.9)		**0.79 (0.63, 0.98)**	
Smoking status					
Never	7074 (51.4)	816.7 (669.1, 1006.0)	** < 0.001**	0.85 (0.66, 1.08)	0.965
Former	3986 (29.0)	786.3 (644.9, 953.5)		**0.68 (0.50, 0.93)**	
Current	2697 (19.6)	772.3 (632.7, 950.5)		0.90 (0.63, 1.27)	
Drinking status					
Never	1870 (14.7)	815.4 (675.6, 1020.8)	**0.002**	0.74 (0.54, 1.01)	0.727
Former	2795 (21.9)	805.2 (647.6, 988.4)		0.77 (0.52, 1.13)	
Current	8088 (63.4)	791.2 (652.6, 968.2)		0.83 (0.69, 1.01)	
Hypertension					
No	6326 (46.0)	806.6 (667.9, 985.9)	** < 0.001**	0.86 (0.67, 1.11)	0.469
Yes	7438 (54.0)	788.0 (642.8, 974.3)		**0.72 (0.59, 0.89)**	
Diabetes mellitus					
No	10,195 (74.1)	798.1 (657.8, 977.3)	0.440	**0.79 (0.66, 0.95)**	0.381
Yes	3560 (25.9)	796.0 (650.8, 987.1)		0.89 (0.60, 1.31)	
CCI					
< 1	5543 (40.3)	801.5 (666.2, 985.4)	**0.020**	0.84 (0.67, 1.06)	0.822
≥ 1	8221 (59.7)	794.3 (648.3, 975.5)		**0.77 (0.61, 0.98)**	
eGFR, mL/min/1.73 m^2^					
< 60	1345 (9.8)	704.7 (573.4, 871.3)	** < 0.001**	0.67 (0.43, 1.05)	0.266
60–90	5777 (42.0)	788.2 (649.7, 969.1)		**0.72 (0.57, 0.92)**	
≥ 90	6637 (48.2)	821.9 (676.2, 1014.9)		0.88 (0.70, 1.11)	
Energy intake, kcal					
< 1850	5799 (42.1)	791.7 (651.8, 983.5)	0.680	0.84 (0.67, 1.05)	0.557
≥ 1850	5808 (42.2)	800.0 (659.6, 976.5)		**0.78 (0.63, 0.97)**	

The odds ratio (95% confidence interval) was calculated in the continuous model adjusting age, sex, race/ethnicity, IPR, BMI, smoking and drinking status, hypertension, diabetes mellitus, CCI, eGFR, and energy intake

*Abbreviations*: *SE* Standard error, *BMI* Body mass index, *IPR* Family income to poverty ratio, *GED* General Educational Development, *CCI* Charlson Comorbidity Index; eGFR, estimated glomerular filtration rate

### Serum Klotho and hyperlipidemia prevalence

**Fig. 1:**
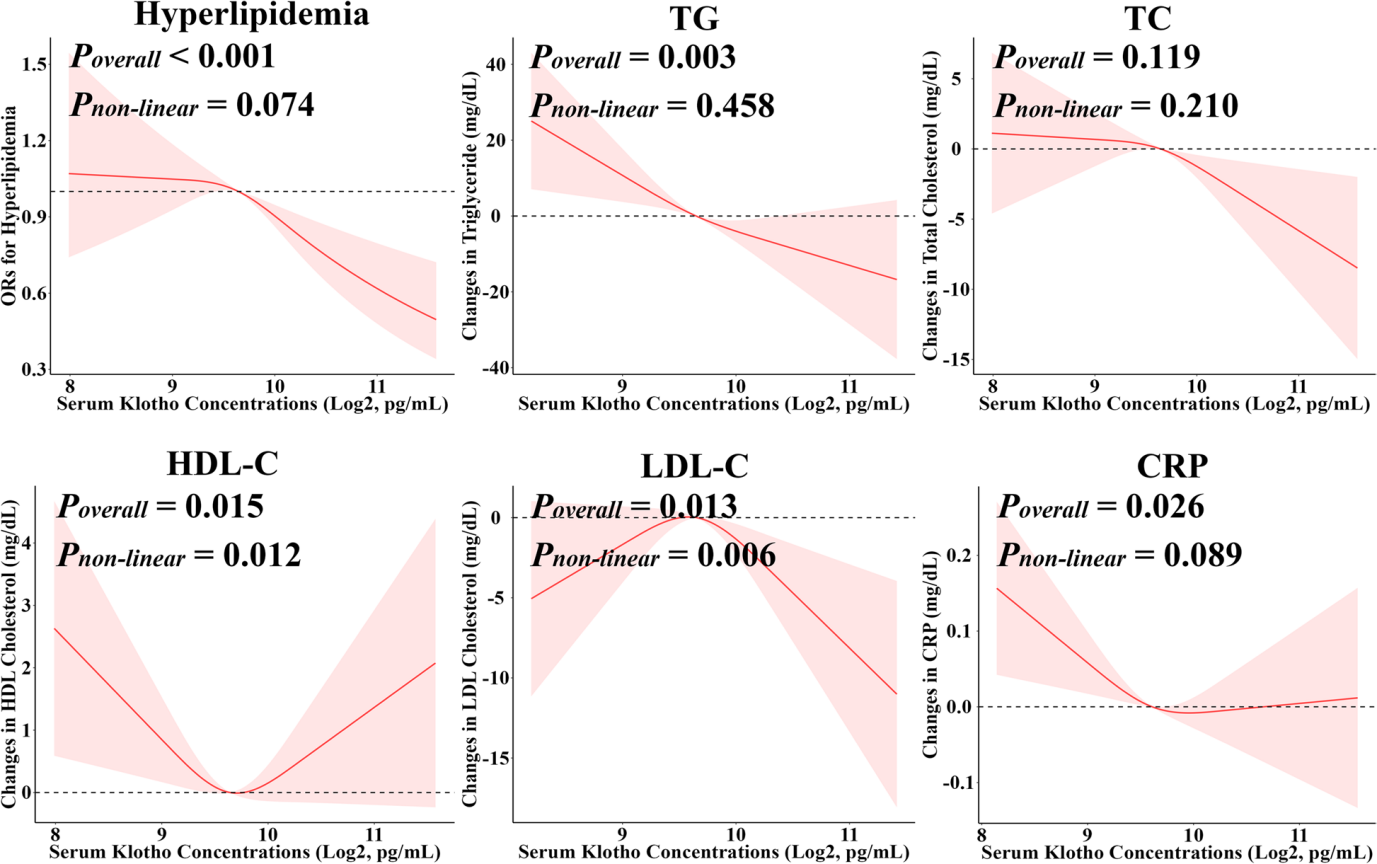
The dose–response relationships of Serum Klotho concentrations with hyperlipidemia prevalence, plasma lipid and serum C-reaction protein levels. Abbreviations: TG, Triglycerides; TC, Total cholesterol; HDL-C, High-density lipoprotein cholesterol; LDL-C, Low-density lipoprotein cholesterol; CRP, C-reaction protein. Models were adjusted for age, sex, race/ethnicity, BMI, IPR, educational attainment, smoking and drinking status, hypertension, diabetes, CCI, eGFR, and energy intake

Serum Klotho concentrations displayed a negative association with hyperlipidemia risk, even after accounting for various confounding factors (Table 3). Individuals in the fourth and fifth quintile of serum Klotho respectively had adjusted odds ratios (OR) of 0.77 (95%CI: 0.65, 0.93) and 0.67 (95%CI: 0.65, 0.93) for hyperlipidemia, with a significant linear trend (*P*_trend_ < 0.001). Doubling of serum Klotho concentrations was linked to a 0.81-fold (95%CI: 0.68, 0.95) decreased adjusted risk of hyperlipidemia. Besides, a significant monotonic dose–response curve between serum Klotho and hyperlipidemia risk was established in the RCS model (*P*_overall_ < 0.001, *P*_non-linear_ = 0.074) (Fig. 1).

**Table 3:** The association of serum Klotho concentrations with hyperlipidemia prevalence and plasma lipid levels, NHANES 2007–2015

Outcomes	Categorical models						Continuous models	
	Quintile 1 (< 620 pg/mL)	Quintile 2 (620.0–742.6 pg/mL)	Quintile 3 (742.7–867.4 pg/mL)	Quintile 4 (867.5–1053.6 pg/mL)	Quintile 5 (> 1053.6 pg/mL)	*P*_trend_	Doubling change	*P* value
Hyperlipidemia								
Model 1	1.00 (ref.)	1.03 (0.86, 1.24)	0.92 (0.76, 1.11)	**0.77 (0.65, 0.93)**	**0.67 (0.56, 0.80)**	** < 0.001**	**0.76 (0.67, 0.86)**	** < 0.001**
Model 2	1.00 (ref.)	1.05 (0.86, 1.27)	0.95 (0.78, 1.16)	**0.81 (0.67, 0.97)**	**0.73 (0.61, 0.88)**	** < 0.001**	**0.81 (0.71, 0.92)**	**0.002**
Model 3	1.00 (ref.)	1.15 (0.92, 1.45)	0.99 (0.78, 1.25)	**0.79 (0.64, 0.98)**	**0.76 (0.61, 0.96)**	** < 0.001**	**0.81 (0.68, 0.95)**	**0.011**
TG								
Model 1	0.00 (ref.)	–10.30 (–23.25, 2.65)	–8.85 (–21.87, 4.18)	**–19.92 (–33.03, –6.82)**	**–21.56 (–33.82, –9.3)**	** < 0.001**	**–15.51 (–24.00, –7.02)**	** < 0.001**
Model 2	0.00 (ref.)	–11.47 (–24.52, 1.58)	–9.65 (–22.95, 3.64)	**–20.37 (–33.72, –7.02)**	**–19.61 (–32.36, –6.86)**	**0.002**	**–14.04 (–22.99, –5.09)**	**0.003**
Model 3	0.00 (ref.)	–7.07 (–21.12, 6.97)	–6.50 (–19.69, 6.70)	**–19.70 (–32.46, –6.94)**	**–17.05 (–30.62, –3.48)**	**0.005**	**–13.25 (–22.47, –4.02)**	**0.007**
TC								
Model 1	0.00 (ref.)	–0.85 (–4.62, 2.92)	0.05 (–3.86, 3.97)	–1.44 (–5.01, 2.13)	–1.23 (–4.95, 2.49)	0.450	–1.88 (–4.18, 0.43)	0.115
Model 2	0.00 (ref.)	–1.09 (–4.89, 2.72)	–0.58 (–4.43, 3.27)	–2.20 (–5.74, 1.35)	–2.68 (–6.32, 0.96)	0.100	**–3.07 (–5.33, –0.81)**	**0.009**
Model 3	0.00 (ref.)	–0.46 (–4.43, 3.51)	–0.50 (–4.60, 3.59)	–2.50 (–6.59, 1.60)	–1.04 (–5.65, 3.57)	0.482	–2.57 (–5.51, 0.36)	0.091
HDL-C								
Model 1	0.00 (ref.)	**–1.39 (–2.74, –0.04)**	–0.87 (–2.04, 0.31)	0.38 (–0.87, 1.64)	0.68 (–0.66, 2.02)	**0.028**	0.50 (–0.45, 1.44)	0.306
Model 2	0.00 (ref.)	–1.09 (–2.32, 0.13)	–0.59 (–1.62, 0.44)	0.59 (–0.62, 1.80)	0.00 (–1.16, 1.16)	0.224	–0.02 (–0.89, 0.84)	0.961
Model 3	0.00 (ref.)	**–1.53 (–2.63, –0.43)**	–0.84 (–2.06, 0.38)	0.03 (–1.27, 1.34)	–0.26 (–1.73, 1.21)	0.512	–0.23 (–1.26, 0.80)	0.664
LDL-C								
Model 1	0.00 (ref.)	3.49 (–0.06, 7.04)	**3.83 (0.23, 7.42)**	1.73 (–2.14, 5.60)	0.83 (–2.97, 4.63)	0.831	–0.29 (–3.11, 2.53)	0.841
Model 2	0.00 (ref.)	3.39 (–0.05, 6.84)	3.34 (–0.16, 6.84)	1.36 (–2.43, 5.14)	–0.06 (–3.85, 3.72)	0.512	–1.01 (–3.82, 1.81)	0.487
Model 3	0.00 (ref.)	**3.76 (0.31, 7.21)**	2.28 (–1.53, 6.10)	–0.33 (–4.07, 3.41)	0.15 (–4.15, 4.44)	0.435	–1.53 (–4.66, 1.59)	0.340

Model 1 did not adjust any potential confounders. Model 2 adjusted for inherent demographic factors including age, sex, and race/ethnicity. Model 3 further adjusted for BMI, IPR, educational attainment, smoking and drinking status, hypertension, diabetes, hypertension, diabetes, CCI, eGFR, and energy intake

*Abbreviations*: *TG* Triglycerides, *TC* Total cholesterol, *HDL-C* High-density lipoprotein cholesterol, *LDL-C* Low-density lipoprotein cholesterol

### Serum Klotho and plasma lipid concentrations

In the linear regression model, compared with the reference group of serum Klotho, individuals in the fourth and fifth quintile respectively had a 19.70 (95%CI: 6.94, 32.46) and 17.05 (95%CI: 3.48, 30.62) mg/dL decreased TG levels, with significant linear trend (*P*_trend_ = 0.005) (Table 2). The RCS model demonstrated a negative linear dose–response relationship between serum Klotho and plasma TG levels (*P*_overall_ = 0.003, *P*_non-linear_ = 0.458). Besides, doubling of serum Klotho concentrations was respectively associated with a 13.25 (95%CI: 4.02, 22.47) mg/dL and 2.57 (–0.36, 5.51) mg/dL decreased TG and TC levels. Intriguingly, significant “U” shape and inverted “U” shape dose–response curves of serum Klotho with plasma HDL-C and LDL-C levels were respectively established (Fig. 1).

### Subgroup analysis

Subgroup and interaction analyses were performed to explore the influence of age, sex, race/ethnicity, and other relevant factors on the relationship between serum Klotho concentrations and the risk of hyperlipidemia (Table 2). Serum Klotho concentrations were linked to decreased hyperlipidemia risk in elder, female, Non-Hispanic black, and participants with high BMI and educational level. No significant modification effects of the above-mentioned stratification factors were suggested.

### Mediation analysis

As shown in Table 4, compared to individuals in the first quintile of serum Klotho, those in the third, fourth, and fifth quintile experienced a decrease in CRP levels of 0.07 (95%CI: 0.002, 0.13), 0.08 (95%CI: 0.02, 0.15), and 0.05 (95%CI: –0.03, 0.12) mg/dL, respectively. This decrease showed a marginally significant trend (*P*_trend_ = 0.05). Doubling of serum Klotho concentrations was linked with a 0.05 (95%CI: 0.001, 0.09) mg/dL decreased CRP levels. However, we observed no significant mediation effects of CRP on the relationships between serum Klotho concentrations and the prevalence of hyperlipidemia, as well as plasma lipid levels (Table 5).

**Table 4:** The association of serum soluble alpha-klotho concentrations with C-reaction protein levels, NHANES 2007–2015

Outcomes	Categorical models						Continuous models	
	Quintile 1	Quintile 2	Quintile 3	Quintile 4	Quintile 5	*P*_trend_	Doubling change	*P* value
Model 1	1.00 (ref.)	–0.02 (–0.1, 0.07)	**–0.08 (–0.15, –0.02)**	**–0.11 (–0.18, –0.05)**	–0.06 (–0.13, 0.02)	**0.038**	**–0.05 (–0.10, –0.001)**	**0.043**
Model 2	1.00 (ref.)	–0.01 (–0.1, 0.07)	**–0.08 (–0.15, –0.01)**	**–0.11 (–0.17, –0.05)**	–0.07 (–0.15, 0.007)	**0.011**	**–0.06 (–0.11, –0.02)**	**0.011**
Model 3	1.00 (ref.)	0.01 (–0.07, 0.09)	**–0.07 (–0.13, –0.002)**	**–0.08 (–0.15, –0.02)**	–0.05 (–0.12, 0.03)	0.050	**–0.05 (–0.09, –0.001)**	**0.044**

Model 1 did not adjust any potential confounders. Model 2 adjusted for inherent demographic factors including age, sex, and race/ethnicity. Model 3 further adjusted for BMI, IPR, educational attainment, smoking and drinking status, hypertension, diabetes, hypertension, diabetes, CCI, eGFR, and energy intake

**Table 5:** Mediating effects of C-reaction protein (CRP) levels on the associations of serum Klotho concentrations with hyperlipidemia prevalence and plasma lipid levels, NHANES 2007–2015

Outcomes	Total effects	Direct effects	Proportion mediated by CRP (*P* value)
	OR/Coefficient (95%CI) ^a^	OR/Coefficient (95%CI) ^b^	
Hyperlipidemia	**0.81 (0.68, 0.95)**	**0.82 (0.71, 0.94)**	0.59% (*P* = 0.250)
TG	**–13.25 (–22.47, –4.02)**	**–8.41 (–16.27, –0.54)**	0.90% (*P* = 0.478)
TC	–2.57 (–5.51, 0.36)	**–2.55 (–4.69, –0.41)**	2.49% (*P* = 0.124)
HDL-C	–0.23 (–1.26, 0.80)	**–**0.10 (**–**0.86, 0.65)	-0.44% (*P* = 0.980)
LDL-C	–1.53 (–4.66, 1.59)	**–**0.96 (**–**3.65, 1.72)	6.72% (*P* = 0.480)

*Abbreviations*: *TG* Triglycerides, *TC* Total cholesterol, *HDL-C* High-density lipoprotein cholesterol, *LDL-C* Low-density lipoprotein cholesterol

^a^Total effects of serum Klotho concentrations with hyperlipidemia prevalence and plasma lipid levels were estimated with adjusting for age, sex, race/ethnicity, BMI, IPR, educational attainment, smoking and drinking status, hypertension, diabetes, CCI, eGFR, and energy intake

^b^Direct effects of serum Klotho concentrations with hyperlipidemia prevalence and plasma lipid levels were estimated with adjusting for age, sex, race/ethnicity, BMI, IPR, educational attainment, smoking and drinking status, hypertension, diabetes, CCI, eGFR, energy intake, and C-reaction protein (CRP) levels

### Sensitivity analysis

The significant associations between serum Klotho concentrations and hyperlipidemia prevalence as well as plasma lipid levels remained robust in most sensitivity analyses (Supplementary Table S1-S3). The method of excluding participants with extreme serum Klotho concentrations, extreme BMI levels, or CCI ≥ 5 caused no substantial changes while excluding individuals under anti-hyperlipidemic treatment weakened the beneficial effects of Klotho on hyperlipidemia prevalence and plasma TG levels but strengthened its effects on reducing plasma TC concentrations.

## Discussion

In a nationally representative sample of the U.S. adults, serum Klotho concentrations were negatively associated with hyperlipidemia risk and plasma TG levels, with monotonic dose–response relationships. The beneficial effect of Klotho on hyperlipidemia control was more prominent in elder, female, Non-Hispanic black, and adults with high educational levels. Besides, higher serum Klotho concentrations were associated with lower CRP levels, yet no substantial mediation effect of CRP on the relationships of serum Klotho concentrations with the prevalence of hyperlipidemia and plasma lipid levels were observed.

In the present study, we conducted a comparison of serum Klotho concentrations to those reported in previous studies. The serum Klotho concentrations (25th: 656.5 pg/mL, median: 797.6 pg/mL, 75th: 979.5 pg/mL) in our population were comparable to those found in adults from Italy (median: 669 pg/mL) [33], Mexica (mean: 744 pg/mL) [34], Japan (mean: 616.3 pg/mL) [35], and community-dwelling adults from Pennsylvania, USA (median: 709.9 pg/mL) [36]; but were notably higher than that in adults from China (median: 381.8 pg/mL) [37] and were lower than that in healthy men from Poland (mean: 1144.38 pg/mL) [38]. Several factors including genetic inheritance, circadian variations, dietary patterns, and physical condition impact the characteristics of serum Klotho [19, 39]. The comparison revealed variations in serum Klotho concentrations across regions, ethnicities, and lifestyles.

The beneficial effects of Klotho on plasma lipid control, especially on the TG level were observed in the study. Kanako et al. built a mice model that only expressing βKlotho in hepatocytes [14]. They demonstrated that βKlotho in hepatocytes was necessary for lipid and bile acid homeostasis [14]. After administrating αKlotho peripherally for 5 weeks in high-fat diet-induced mice, Rao et al. observed reduced lipid accumulation in liver and adipose tissue [15]. Gu et al. demonstrated that sKlotho could ameliorate lipid accumulation via inhibiting the PI3K/AKT signaling [40]. However, Kobayashi et al. found no beneficial effects of Klotho on atherosclerosis and plasma TC levels in experimental rodent models [17]. In a cross-sectional study, an inverse relationship between sKlotho concentrations and the cardiometabolic risk score was only observed in middle-aged adults [41]. Dongiovanni et al. described an association between βKlotho gene variation and liver damage, specifically involving ballooning and lobular inflammation, in pediatric NAFLD [16]. Their research group found increased lipid accumulation and up-regulation of lipotoxic and pro-inflammatory genes after down-regulating the expression of Klotho protein in hepatocytes [16]. Besides, Cheng et al. established a negative association between serum Klotho concentrations and metabolic syndrome components including high TG levels [18]. Meanwhile, Lee et al. noticed that high circulating Klotho concentrations were linked with improved cardiovascular disease risk factors including TG and TC levels [19]. However, in a small sample study (*n* = 186), Żelaźniewicz found no association between Klotho concentrations and cardiometabolic risk factors including TC levels [38]. Considering the biological role of Klotho, our findings along with additional studies provided further evidence supporting the potential beneficial impact of Klotho on plasma lipid control.

βKlotho functions as a co-receptor for FGF21, facilitating various biological processes such as cell proliferation and differentiation, angiogenesis and tissue regeneration, oxidative stress and inflammatory response, glucose and lipid metabolism [42]. However, the associations between FGF21 and metabolic diseases such as NAFLD, atherosclerosis, and hyperlipidemia have still not reached an agreement [21, 42]. Treatment with recombinant FGF21 in diet-induced obese mice and other animal models reduced TG levels and ameliorated hepatic steatosis via inhibiting the lipogenic gene expression (*srebp-1*), stimulating brown adipose tissue and browning white adipose tissue, and improving insulin resistance [22, 43]. In a randomized, placebo-controlled trial, Gaich et al. first reported the lipid-lowering effect of FGF21 including decreased levels of TG and LDL-C, increased levels of HDL-C, and a transition to a less atherogenic apolipoprotein profile [44]. However, positive associations of FGF21 with NAFLD risk and TG levels also were suggested in some cross-sectional and prospective studies [45, 46]. Despite the pharmacological effects of FGF21 in humans is now a tantalizing and exciting prospect, more clinical trials and long-term prospective studies are required to bolster its therapeutic applications.

Despite the exact mechanism of how Klotho improves plasma lipid levels is unclear, its anti-oxidative and anti-inflammatory properties might play a vital role. Klotho deficiency enhances reactive oxygen species production and aggravates oxidative stress [47]. Klotho administration preserves mitochondrial function and mitigates oxidative stress via promoting FoxO and Nox2 protein expression, activating the cellular protective Nrf2 pathway, and regulating AMPK/mTOR pathway [48, 49]. Klotho could exert the anti-inflammatory effect via regulating NF-κB and NLRP3-inflammasome activation, inducing proteolytic degradation of TLR4, inhibiting TNF-α responses on the strengthening of inflammatory processes, and suppressing subsequent production of pro-inflammatory cytokines [13, 50, 51]. Apolipoprotein E (apoE), a major apolipoprotein involved in lipoprotein metabolism, is closely associated with lipid levels, cardiovascular risk, and Alzheimer disease. Studies have indicated that apoE deficient animals could spontaneously develop severe dyslipidemia and liver steatosis [52]. However, some studies found that Klotho-VS heterozygosity could protect against APOE4-associated diseases [53]. Moreover, the positive impact of Klotho on obesity, diabetes, hypertension, arteriosclerosis, renal and liver function, metabolic homeostasis, and gut microbiota could contribute to its hypolipidemic effects [7, 12, 54].

Sex difference existed in the association between Klotho concentration and hyperlipidemia risk. Klotho exerted more beneficial effects on plasma lipid control in females than males. Females usually have a greater genetic expression of antioxidant genes and enzymatic activity compared to males, which might have an additive or synergistic effects with Klotho [55]. Besides, male participants exhibited a high prevalence of smoking and drinking, these unfavorable factors might neutralize Klotho’s beneficial effects on plasma lipid control. Theoretically, Klotho expression level decreases with age, BMI, and renal function [56]. However, we observed negative associations of Klotho concentrations with hyperlipidemia risk among the elderly and individuals with high BMI levels. Further research is imperative to elucidate the precise biological mechanisms behind the varying impacts of Klotho observed in specific groups, as these differences may stem from a range of comprehensive factors.

We specifically explored the relationship between Klotho concentrations and the prevalence of hyperlipidemia, as well as plasma lipid levels. Our study strengths encompassed standardized quality controls in laboratory analysis and covariates collection, national representativeness of the subjects, and multiple sensitivity analyses. Nevertheless, the cross-sectional design imposes restrictions, preventing the establishment of causal relationships. However, considering the therapeutic benefit of the FGF-Klotho endocrine axes in multiple systems, it’s more reasonable to speculate that Klotho improves plasma lipid levels, not vice versa. Secondly, despite serum Klotho is a recommended biomarker that has been extensively utilized in numerous epidemiological studies, its concentrations might not sufficiently reflect Klotho protein in tissue and are affected by circadian rhythm and temporal variation [56, 57]. However, there presently lacks evidence to support any available biological matrix as a dependable biomarker of Klotho protein. Thirdly, although we accounted for various potential confounding factors, unmeasured and unknown factors such as klotho methylation status might still play a role in the associations. Given the consistent findings observed across various statistical models and sensitivity analyses, causes overrode random occurrences in the relationships.

## Conclusions

Klotho concentrations exhibited an inverse association with the risk of hyperlipidemia and plasma TG levels among nationally representative U.S. adults. The beneficial effects of Klotho to some extent was ascribed to its anti-oxidative and anti-inflammatory properties. Our findings emphasize an important public health concern and offer new perspectives on the clinical implications of Klotho in regulating plasma lipid levels. Further studies are recommended to confirm the causal relationship and elucidate the biological mechanisms behind the foregoing association.

### Supplementary Information

**Additional file 1: Table S1.** The association of serum Klotho concentrations with hyperlipidemia prevalence and plasma lipid levels, after excluding extreme blood Mn concentrations (outside the range of mean±3×standard deviation). **Table S2.** The association of serum Klotho concentrations with hyperlipidemia prevalence and plasma lipid levels, after excluding participants with extreme BMI levels (< 15, or ≥ 40 kg/m^2^) or CCI ≥ 5. **Table S3.** The association of serum Klotho concentrations with hyperlipidemia prevalence and plasma lipid levels, after excluding individuals under anti-hyperlipidemic treatment. **Table S4.** Characteristics of the study population by sex, NHANES 2007-2015.

## Data Availability

The datasets presented in this study can be found in online repositories (https://www.cdc.gov/nchs/nhanes/index.htm).

## Abbreviations

BMI: Body mass index
CDC: Centers for Disease Control and Prevention
CCI: Charlson Comorbidity Index
CI: Confidence interval
CRP: C-reaction protein
eGFR: Estimated glomerular filtration rate
HDL-C: High-density lipoprotein cholesterol
IPR: Income-to-poverty ratio
IHD: Ischemic heart disease
LDL-C: Low-density lipoprotein cholesterol
mKlotho: Membrane-bound Klotho
MEC: Mobile examination center
NCHS: National Center for Health Statistics
NHANES: National Health and Nutrition Examination Survey
NAFLD: Nonalcoholic fatty liver disease
OR: Odds ratio
RCS: Restricted cubic spline
sKlotho: Soluble Klotho
TC: Total cholesterol
TG: Triglyceride
