# Supplementary material for: The association of serum Klotho concentrations with hyperlipidemia prevalence and lipid levels among US adults: a cross-sectional study
Source: BMC Public Health. 2023 Aug 28;23:1645. doi: 10.1186/s12889-023-16566-y (PMC10463308; doi:10.1186/s12889-023-16566-y)
Supplement: Supplementary file 1 — Additional file 1: Table S1. The association of serum Klotho concentrations with hyperlipidemia prevalence and plasma lipid levels, after excluding extreme blood Mn concentrations (outside the range of mean±3×standard deviation). Table S2. The association of serum Klotho concentrations with hyperlipidemia prevalence and plasma lipid levels, after excluding participants with extreme BMI levels (< 15, or ≥ 40 kg/m2) or CCI ≥ 5. Table S3. The association of serum Klotho concentrations with hyperlipidemia prevalence and plasma lipid levels, after excluding individuals under anti-hyperlipidemic treatment. Table S4. Characteristics of the study population by sex, NHANES 2007-2015. [file 12889_2023_16566_MOESM1_ESM.docx]

**The association of serum** **Klotho concentrations with** **hyperlipidemia prevalence and lipid levels among US adults: A** **cross-sectional study**

Shunli Jiang^a,*, 1^, Yongxin Wang^b, 1^, Zengliang Wang^b^, Lu Zhang^c^, Feng Jin^c, *^, Bo Li^c, *^

^a^ Institute of Occupational Health and Environmental Medicine, Department of Public Health, Jining Medical University, Jining, Shandong, China, 272000

^b^ Department of Neurosurgery Center, The First Affiliated Hospital of Xinjiang Medical University, Urumqi, Xinjiang, China

^c^ Department of Neurosurgery, Affiliated Hospital of Jining Medical University, Jining, China

^*^ Corresponding author: utopianjiang@163.com (SL. Jiang); jinfengsdjn@163.com (F. Jin); libo5479937@126.com (B. Li)

^1^ These authors contributed equally to this work.

Address: Department of Public Health, Jining Medical University, #33 Jianshe Road, Rencheng District, Jining, Shan Dong, 272000, China

**Table of Contents**

Table S1. The association of serum Klotho concentrations with hyperlipidemia prevalence and plasma lipid levels, after excluding extreme blood Mn concentrations (outside the range of mean±3×standard deviation).

Table S2. The association of serum Klotho concentrations with hyperlipidemia prevalence and plasma lipid levels, after excluding participants with extreme BMI levels (< 15, or ≥ 40 kg/m^2^) or CCI ≥ 5.

Table S3. The association of serum Klotho concentrations with hyperlipidemia prevalence and plasma lipid levels, after excluding individuals under anti-hyperlipidemic treatment.

Table S4. Characteristics of the study population by sex, NHANES 2007-2015.

| Table S1. The association of serum Klotho concentrations with hyperlipidemia prevalence and plasma lipid levels, after excluding extreme blood Mn concentrations (outside the range of mean±3×standard deviation). | | | | | | | | | |
| --- | --- | --- | --- | --- | --- | --- | --- | --- | --- |
| Outcomes | Categorical models | | | | | |  | Continuous models | |
|  | Quintile 1 | Quintile 2 | Quintile 3 | Quintile 4 | Quintile 5 | *P*_trend_ |  | Doubling change | *P* value |
| Hyperlipidemia | |  |  |  |  |  |  |  |  |
| Model 1 | 1.00 (ref.) | 1.03 (0.86, 1.24) | 0.92 (0.76, 1.11) | **0.77 (0.65, 0.92)** | **0.66 (0.55, 0.79)** | **< 0.001** |  | **0.73 (0.63, 0.84)** | **< 0.001** |
| Model 2 | 1.00 (ref.) | 1.05 (0.87, 1.26) | 0.95 (0.78, 1.15) | **0.81 (0.67, 0.97)** | **0.72 (0.59, 0.86)** | **< 0.001** |  | **0.78 (0.67, 0.90)** | **< 0.001** |
| Model 3 | 1.00 (ref.) | 1.15 (0.92, 1.44) | 0.99 (0.78, 1.24) | **0.79 (0.64, 0.97)** | **0.75 (0.60, 0.95)** | **< 0.001** |  | **0.78 (0.65, 0.93)** | **0.007** |
| TG |  |  |  |  |  |  |  |  |  |
| Model 1 | 0.00 (ref.) | –10.3 (–23.25, 2.65) | –8.85 (–21.87, 4.18) | **–19.92 (–33.03, –6.82)** | **–20.78 (–33.08, –8.48)** | **<0.001** |  | **–15.67 (–25.00, –6.35)** | **0.001** |
| Model 2 | 0.00 (ref.) | –11.49 (–24.53, 1.55) | –9.68 (–22.97, 3.61) | **–20.41 (–33.76, –7.06)** | **–19.04 (–31.78, –6.29)** | **0.003** |  | **–14.43 (–24.16, –4.70)** | **0.005** |
| Model 3 | 0.00 (ref.) | –7.08 (–21.13, 6.97) | –6.53 (–19.73, 6.67) | **–19.72 (–32.51, –6.92)** | **–16.96 (–30.5, –3.42)** | **0.005** |  | **–14.29 (–24.25, –4.33)** | **0.007** |
| TC |  |  |  |  |  |  |  |  |  |
| Model 1 | 0.00 (ref.) | –0.85 (–4.62, 2.92) | 0.05 (–3.86, 3.97) | –1.44 (–5.01, 2.13) | –0.44 (–4.12, 3.24) | 0.748 |  | –0.88 (–3.26, 1.50) | 0.471 |
| Model 2 | 0.00 (ref.) | –1.10 (–4.90, 2.71) | –0.60 (–4.44, 3.24) | –2.22 (–5.76, 1.32) | –1.95 (–5.54, 1.64) | 0.212 |  | –2.20 (–4.50, 0.10) | 0.065 |
| Model 3 | 0.00 (ref.) | –0.47 (–4.44, 3.49) | –0.53 (–4.62, 3.55) | –2.53 (–6.61, 1.56) | –0.31 (–4.78, 4.17) | 0.674 |  | –1.69 (–4.57, 1.20) | 0.257 |
| HDL |  |  |  |  |  |  |  |  |  |
| Model 1 | 0.00 (ref.) | **–1.39 (–2.74, –0.04)** | –0.87 (–2.04, 0.31) | 0.38 (–0.87, 1.64) | 0.65 (–0.78, 2.08) | **0.043** |  | 0.49 (–0.59, 1.57) | 0.377 |
| Model 2 | 0.00 (ref.) | –1.10 (–2.33, 0.13) | –0.60 (–1.63, 0.43) | 0.58 (–0.63, 1.79) | –0.02 (–1.28, 1.23) | 0.258 |  | –0.01 (–0.98, 0.95) | 0.977 |
| Model 3 | 0.00 (ref.) | –1.53 (–2.63, –0.44) | –0.85 (–2.06, 0.37) | 0.03 (–1.28, 1.33) | –0.23 (–1.77, 1.30) | 0.512 |  | –0.18 (–1.35, 1.00) | 0.771 |
| LDL |  |  |  |  |  |  |  |  |  |
| Model 1 | 0.00 (ref.) | 3.49 (–0.06, 7.04) | 3.83 (0.23, 7.42) | 1.73 (–2.14, 5.60) | 1.49 (–2.2, 5.18) | 0.890 |  | 0.78 (–1.98, 3.53) | 0.582 |
| Model 2 | 0.00 (ref.) | 3.38 (–0.06, 6.82) | 3.32 (–0.18, 6.82) | 1.35 (–2.44, 5.13) | 0.57 (–3.12, 4.26) | 0.742 |  | –0.01 (–2.76, 2.74) | 0.993 |
| Model 3 | 0.00 (ref.) | 3.75 (0.31, 7.19) | 2.24 (–1.57, 6.05) | –0.37 (–4.11, 3.38) | 0.76 (–3.27, 4.79) | 0.582 |  | –0.66 (–3.53, 2.21) | 0.652 |
| Model 1 did not adjust any potential confounders. Model 2 adjusted for inherent demographic factors including age, sex, and race/ethnicity. Model 3 further adjusted for BMI, IPR, educational attainment, smoking and drinking status, hypertension, diabetes, CCI, eGFR, and energy intake. | | | | | | | | | |

| Table S2. The association of serum Klotho concentrations with hyperlipidemia prevalence and plasma lipid levels, after excluding participants with extreme BMI levels (< 15, or ≥ 40 kg/m^2^) or CCI ≥ 5. | | | | | | | | | |
| --- | --- | --- | --- | --- | --- | --- | --- | --- | --- |
| Outcomes | Categorical models | | | | | |  | Continuous models | |
|  | Quintile 1 | Quintile 2 | Quintile 3 | Quintile 4 | Quintile 5 | *P*_trend_ |  | Doubling change | *P* value |
| Hyperlipidemia | |  |  |  |  |  |  |  |  |
| Model 1 | 1.00 (ref.) | 1.05 (0.88, 1.25) | 0.92 (0.76, 1.12) | **0.78 (0.65, 0.93)** | **0.68 (0.57, 0.81)** | **< 0.001** |  | **0.76 (0.67, 0.86)** | **< 0.001** |
| Model 2 | 1.00 (ref.) | 1.06 (0.88, 1.27) | 0.95 (0.78, 1.15) | **0.81 (0.67, 0.96)** | **0.74 (0.61, 0.89)** | **< 0.001** |  | **0.82 (0.72, 0.92)** | **0.002** |
| Model 3 | 1.00 (ref.) | 1.16 (0.93, 1.45) | 0.98 (0.77, 1.24) | **0.79 (0.64, 0.97)** | **0.77 (0.61, 0.97)** | **0.001** |  | **0.81 (0.69, 0.96)** | **0.014** |
| TG |  |  |  |  |  |  |  |  |  |
| Model 1 | 0.00 (ref.) | –8.81 (–20.72, 3.10) | –8.38 (–21.58, 4.83) | **–19.85 (–32.72, –6.97)** | **–22.31 (–34.58, –10.03)** | **<0.001** |  | **–16.27 (–24.7, –7.85)** | **<0.001** |
| Model 2 | 0.00 (ref.) | –9.84 (–21.72, 2.04) | –9.29 (–22.68, 4.11) | **–20.25 (–33.30, –7.19)** | **–20.26 (–33.08, –7.44)** | **0.001** |  | **–14.74 (–23.63, –5.86)** | **0.002** |
| Model 3 | 0.00 (ref.) | –5.41 (–18.12, 7.30) | –5.74 (–19.46, 7.98) | **–18.52 (–31.41, –5.63)** | **–16.39 (–29.84, –2.94)** | **0.005** |  | **–12.89 (–22.1, –3.69)** | **0.008** |
| TC |  |  |  |  |  |  |  |  |  |
| Model 1 | 0.00 (ref.) | –0.54 (–3.84, 2.75) | –0.43 (–4.21, 3.36) | –1.64 (–5.12, 1.83) | –1.90 (–5.46, 1.67) | 0.219 |  | **–2.60 (–4.83, –0.37)** | **0.025** |
| Model 2 | 0.00 (ref.) | –0.60 (–3.97, 2.78) | –0.78 (–4.52, 2.97) | –2.14 (–5.61, 1.34) | –3.02 (–6.57, 0.52) | 0.054 |  | **–3.54 (–5.76, –1.32)** | **0.003** |
| Model 3 | 0.00 (ref.) | 0.81 (–2.69, 4.31) | –0.26 (–4.17, 3.66) | –1.92 (–5.85, 2.00) | –0.75 (–5.06, 3.56) | 0.435 |  | –2.62 (–5.42, 0.18) | 0.072 |
| HDL |  |  |  |  |  |  |  |  |  |
| Model 1 | 0.00 (ref.) | **–1.56 (–2.88, –0.24)** | –1.15 (–2.38, 0.09) | 0.30 (–1.04, 1.63) | 0.52 (–0.86, 1.89) | **0.046** |  | 0.34 (–0.65, 1.33) | 0.506 |
| Model 2 | 0.00 (ref.) | **–1.23 (–2.41, –0.04)** | –0.78 (–1.86, 0.31) | 0.53 (–0.74, 1.81) | –0.15 (–1.36, 1.07) | 0.311 |  | –0.19 (–1.09, 0.72) | 0.691 |
| Model 3 | 0.00 (ref.) | **–1.51 (–2.60, –0.42)** | –0.96 (–2.21, 0.29) | –0.01 (–1.41, 1.39) | –0.35 (–1.87, 1.16) | 0.613 |  | –0.36 (–1.45, 0.73) | 0.517 |
| LDL |  |  |  |  |  |  |  |  |  |
| Model 1 | 0.00 (ref.) | **3.50 (0.06, 6.94)** | 3.07 (–0.65, 6.79) | 1.41 (–2.78, 5.60) | –0.43 (–4.47, 3.61) | 0.407 |  | –1.18 (–4.12, 1.76) | 0.432 |
| Model 2 | 0.00 (ref.) | **3.54 (0.19, 6.89)** | 2.77 (–0.89, 6.43) | 1.20 (–2.91, 5.30) | –1.09 (–5.10, 2.92) | 0.248 |  | –1.73 (–4.66, 1.20) | 0.252 |
| Model 3 | 0.00 (ref.) | **3.92 (0.39, 7.46)** | 1.56 (–2.44, 5.56) | –0.53 (–4.62, 3.57) | –0.62 (–5.18, 3.95) | 0.262 |  | –2.03 (–5.31, 1.24) | 0.228 |
| Model 1 did not adjust any potential confounders. Model 2 adjusted for inherent demographic factors including age, sex, and race/ethnicity. Model 3 further adjusted for BMI, IPR, educational attainment, smoking and drinking status, hypertension, diabetes, CCI, eGFR, and energy intake. | | | | | | | | | |

| Table S3. The association of serum Klotho concentrations with hyperlipidemia prevalence and plasma lipid levels, after excluding individuals under anti-hyperlipidemic treatment. | | | | | | | | | |
| --- | --- | --- | --- | --- | --- | --- | --- | --- | --- |
| Outcomes | Categorical models | | | | | |  | Continuous models | |
|  | Quintile 1 | Quintile 2 | Quintile 3 | Quintile 4 | Quintile 5 | *P*_trend_ |  | Doubling change | *P* value |
| Hyperlipidemia | |  |  |  |  |  |  |  |  |
| Model 1 | 1.00 (ref.) | 1.05 (0.85, 1.29) | 1.00 (0.82, 1.23) | **0.84 (0.70, 1.02)** | **0.79 (0.65, 0.95)** | **< 0.001** |  | **0.85 (0.74, 0.97)** | **0.019** |
| Model 2 | 1.00 (ref.) | 1.05 (0.85, 1.30) | 1.00 (0.81, 1.22) | **0.84 (0.70, 1.02)** | **0.80 (0.66, 0.97)** | **0.002** |  | **0.86 (0.75, 0.98)** | **0.027** |
| Model 3 | 1.00 (ref.) | 1.15 (0.89, 1.49) | 1.03 (0.81, 1.30) | **0.81 (0.65, 1.01)** | 0.84 (0.65, 1.07) | **0.016** |  | 0.85 (0.72, 1.01) | 0.075 |
| TG |  |  |  |  |  |  |  |  |  |
| Model 1 | 0.00 (ref.) | –5.56 (–19.99, 8.88) | –6.52 (–20.07, 7.04) | **–15.46 (–29.81, –1.11)** | **–19.49 (–32.85, –6.13)** | **0.001** |  | **–13.81 (–22.38, –5.23)** | **0.002** |
| Model 2 | 0.00 (ref.) | –7.19 (–21.57, 7.20) | –7.17 (–20.99, 6.66) | **–15.69 (–30.22, –1.15)** | **–16.86 (–30.51, –3.20)** | **0.009** |  | **–11.58 (–20.56, –2.60)** | **0.014** |
| Model 3 | 0.00 (ref.) | 1.28 (–12.5, 15.07) | –2.63 (–14.59, 9.32) | **–13.56 (–25.92, –1.19)** | –9.66 (–22.05, 2.73) | **0.030** |  | **–8.58 (–16.88, –0.27)** | **0.047** |
| TC |  |  |  |  |  |  |  |  |  |
| Model 1 | 0.00 (ref.) | –1.01 (–5.28, 3.25) | –3.32 (–7.38, 0.75) | –3.67 (–7.88, 0.55) | **–6.25 (–10.71, –1.78)** | **0.002** |  | **–5.63 (–8.41, –2.84)** | **<0.001** |
| Model 2 | 0.00 (ref.) | –0.75 (–5.11, 3.62) | –3.14 (–7.24, 0.96) | –3.22 (–7.43, 1.00) | **–5.57 (–10.00, –1.14)** | **0.005** |  | **–5.12 (–7.85, –2.38)** | **<0.001** |
| Model 3 | 0.00 (ref.) | 0.26 (–4.68, 5.20) | –2.22 (–7.13, 2.69) | –2.98 (–8.24, 2.28) | –3.02 (–8.80, 2.76) | 0.180 |  | **–3.95 (–7.62, –0.28)** | **0.039** |
| HDL |  |  |  |  |  |  |  |  |  |
| Model 1 | 0.00 (ref.) | **–2.13 (–3.91, –0.35)** | –1.46 (–3.22, 0.30) | 0.60 (–1.16, 2.37) | 0.08 (–1.67, 1.83) | 0.115 |  | 0.23 (–1.08, 1.53) | 0.736 |
| Model 2 | 0.00 (ref.) | –1.44 (–3.09, 0.21) | –0.94 (–2.54, 0.66) | 1.08 (–0.62, 2.79) | –0.39 (–2.03, 1.25) | 0.419 |  | –0.24 (–1.49, 1.02) | 0.713 |
| Model 3 | 0.00 (ref.) | **–2.1 (–3.50, –0.70)** | –1.18 (–3.09, 0.74) | 0.46 (–1.42, 2.33) | –0.53 (–2.54, 1.49) | 0.522 |  | –0.35 (–1.86, 1.16) | 0.651 |
| LDL |  |  |  |  |  |  |  |  |  |
| Model 1 | 0.00 (ref.) | **5.57 (1.66, 9.48)** | 2.29 (–1.53, 6.12) | 1.94 (–2.34, 6.21) | –1.26 (–5.63, 3.11) | 0.135 |  | –2.27 (–5.62, 1.08) | 0.188 |
| Model 2 | 0.00 (ref.) | **5.36 (1.44, 9.28)** | 2.17 (–1.68, 6.02) | 1.99 (–2.25, 6.23) | –0.60 (–5.01, 3.81) | 0.260 |  | –1.07 (–5.08, 1.68) | 0.327 |
| Model 3 | 0.00 (ref.) | **5.91 (1.52, 10.29)** | 2.26 (–1.95, 6.46) | 0.49 (–4.26, 5.24) | 0.77 (–4.49, 6.02) | 0.497 |  | –1.53 (–5.47, 2.42) | 0.451 |
| Model 1 did not adjust any potential confounders. Model 2 adjusted for inherent demographic factors including age, sex, and race/ethnicity. Model 3 further adjusted for BMI, IPR, educational attainment, smoking and drinking status, hypertension, diabetes, CCI, eGFR, and energy intake. | | | | | | | | | |

| Table S4. Characteristics of the study population by sex, NHANES 2007-2015. | | | | |
| --- | --- | --- | --- | --- |
| Characteristics | Total population (n = 13764) | Female (n = 7097) | Male (n = 6667) | *P* value |
| Age, mean (SE), year | 56.2 ± 0.2 | 56.3 ± 0.2 | 56.1 ± 0.2 | 0.200 |
| Race/ethnicity |  |  |  | **0.002** |
| Non-Hispanic black | 2727 (19.8) | 1401 (9.9) | 1326 (8.4) |  |
| Non-Hispanic white | 5920 (43.0) | 2997 (72.4) | 2923 (73.4) |  |
| Mexican American | 2188 (15.9) | 1146 (6.6) | 1042 (6.8) |  |
| Other | 2929 (21.3) | 1553 (11.2) | 1376 (11.4) |  |
| BMI, kg/m^2^ |  |  |  | **< 0.001** |
| <25 | 3607 (27.1) | 2040 (34.2) | 1567 (21.9) |  |
| 25-30 | 4852 (36.4) | 2048 (29.6) | 2804 (44.5) |  |
| ≥30 | 4869 (36.5) | 2714 (36.2) | 2155 (33.6) |  |
| IPR |  |  |  | **< 0.001** |
| <1.30 | 3886 (30.8) | 2092 (19.0) | 1794 (16.4) |  |
| 1.30-3.49 | 4529 (35.9) | 2355 (34.0) | 2174 (31.4) |  |
| ≥3.50 | 4213 (33.4) | 2039 (47.0) | 2174 (52.2) |  |
| Educational level |  |  |  | **0.040** |
| Less than high school | 1876 (13.6) | 941 (6.0) | 935 (6.6) |  |
| High school or GED | 5062 (36.8) | 2554 (31.9) | 2508 (33.3) |  |
| College or above | 6818 (49.6) | 3595 (62.1) | 3223 (60.1) |  |
| Smoking status |  |  |  | **< 0.001** |
| Never | 7074 (51.4) | 4334 (58.0) | 2740 (44.8) |  |
| Former | 3986 (29.0) | 1578 (25.1) | 2408 (34.9) |  |
| Current | 2697 (19.6) | 1182 (16.9) | 1515 (20.3) |  |
| Drinking status |  |  |  | **< 0.001** |
| Never | 1870 (14.7) | 1425 (15.1) | 445 (5.9) |  |
| Former | 2795 (21.9) | 1348 (17.5) | 1447 (18.9) |  |
| Current | 8088 (63.4) | 3750 (67.5) | 4338 (75.2) |  |
| Hypertension |  |  |  | 0.100 |
| No | 6326 (46.0) | 3278 (52.4) | 3048 (50.2) |  |
| Yes | 7438 (54.0) | 3819 (47.6) | 3619 (49.8) |  |
| Diabetes |  |  |  | **< 0.001** |
| No | 10195 (74.1) | 5351 (82.1) | 4844 (78.6) |  |
| Yes | 3560 (25.9) | 1737 (17.9) | 1823 (21.4) |  |
| CCI |  |  |  | **0.010** |
| <1 | 5543 (40.3) | 2772 (41.6) | 2771 (44.5) |  |
| ≥1 | 8221 (59.7) | 4325 (58.4) | 3896 (55.5) |  |
| eGFR, mL/min/1.73 m^2^ |  |  |  | **< 0.001** |
| <60 | 1345 (9.8) | 709 (9.1) | 636 (7.0) |  |
| 60-90 | 5777 (42.0) | 2786 (42.8) | 2991 (46.8) |  |
| ≥90 | 6637 (48.2) | 3598 (48.1) | 3039 (46.2) |  |
| Hyperlipidemia |  |  |  | 0.100 |
| No | 2755 (20.0) | 1307 (19.4) | 1448 (20.8) |  |
| Yes | 11009 (80.0) | 5790 (80.6) | 5219 (79.2) |  |
| Klotho, pg/mL | 797.6 (656.5, 979.5) | 811.6 (660.2,1005.9) | 783.6 (651.6, 957.1) | **< 0.001** |
| TG, mg/dL | 109.0 (76.0, 162.0) | 105.0 (74.0,154.0) | 114.0 (80.0,170.0) | **< 0.001** |
| TC, mg/dL | 198.0 (172.0, 226.0) | 203.0 (178.0,230.0) | 192.0 (165.0,222.0) | **< 0.001** |
| HDL, mg/dL | 51.0 (42.0, 63.0) | 57.0 (47.0,70.0) | 45.0 (38.0,54.0) | **< 0.001** |
| LDL, mg/dL | 116.0 (94.0, 141.0) | 117.0 (96.0,143.0) | 114.0 (91.0,138.0) | **< 0.001** |
| Energy intake, kcal | 1937.5 (1511.5, 2445.5) | 1689.5 (1348.0,2075.5) | 2269.0 (1820.5,2817.0) | **< 0.001** |
| Abbreviations: SE, standard error; BMI, body mass index; IPR, family income to poverty ratio; GED, General Educational Development; CCI, Charlson Comorbidity Index; eGFR, estimated glomerular filtration rate; TG, Triglycerides; TC, Total cholesterol; HDL-C, High-density lipoprotein cholesterol; LDL-C, Low-density lipoprotein cholesterol. Continuous variables were presented as mean (standard error) or median (25th, 75th), according to its distribution; categorical variables were presented as numbers (percentage).  *P*-values were calculated by weighted Student’s t-test, Mann-Whitney *U* test, or chi-square test for different variables. | | | | |
